# Robotic-Assisted vs. Laparoscopic Splenectomy in Children: A Systematic Review and Up-to-Date Meta-Analysis

**DOI:** 10.3390/jpm15110522

**Published:** 2025-11-01

**Authors:** Carlos Delgado-Miguel, Juan Camps, Isabella Garavis Montagut, Ricardo Díez, Javier Arredondo-Montero, Francisco Hernández-Oliveros

**Affiliations:** 1 Department of Pediatric Surgery, Fundación Jiménez Díaz University Hospital, 28040 Madrid, Spain; 2 Institute for Health Research IdiPAZ, La Paz University Hospital, 28046 Madrid, Spain; fhernandezo@salud.madrid.org; 3 Department of Pediatric Surgery, Prisma Health Children’s Hospital, Columbia, SC 29203, USA; juan.camps@prismahealth.org; 4 Research Hotbed in General Surgery and Subspecialties, El Bosque University, Bogotá 110111, Colombia; igaravis@unbosque.edu.co; 5 Department of Pediatric Surgery, Complejo Asistencial Universitario de León, 24008 Castilla y León, Spain; jarredondo@saludcastillayleon.es

**Keywords:** robotic-assisted surgery, robotic surgery, splenectomy, spleen, children, pediatric, minimally invasive surgery

## Abstract

**Introduction**: Robotic splenectomy has emerged as a promising alternative to laparoscopic surgery, offering potential advantages in precision, ergonomics, and individualized surgical planning. In the context of personalized medicine, robotic technology may enable tailoring of surgical strategies to patient-specific anatomy, spleen size, and comorbid hematologic conditions. However, its clinical superiority remains uncertain due to limited and heterogeneous evidence. **Methods**: We performed a systematic review and meta-analysis following PRISMA guidelines, utilizing PubMed, CINAHL, Web of Science, and EMBASE databases to locate studies on robotic splenectomies in children. This review was prospectively registered in PROSPERO (CRD420251104285). Risk of bias was assessed using the ROBINS-I tool for non-randomized studies. Random-effects models were fitted using restricted maximum likelihood (REML), and confidence intervals were adjusted using either Knapp–Hartung (HKSJ) or modified Knapp–Hartung (mKH) methods when appropriate. 95% prediction intervals were calculated, and the certainty of evidence for each outcome was assessed using the GRADE approach. **Results**: This review included 272 pediatric patients from 16 studies conducted between 2003 and 2025, of which five were included in the meta-analysis. No statistically significant differences were observed between robotic and laparoscopic splenectomy for operative time, intraoperative blood loss, conversion to open surgery, blood transfusions, or complications. However, the direction of effect estimates consistently favored the robotic approach. A statistically significant reduction in hospitalization days (−0.93 days; 95% CI: −1.61 to −0.24; *p* = 0.01) was found, though this became marginally significant after HKSJ adjustment (*p* = 0.06). Intraoperative blood loss showed significance in the primary model (−63.88 mL; 95% CI: −120.38 to −7.38; *p* = 0.03), but not after mKH correction (*p* = 0.16). Heterogeneity was substantial-to-extreme for several outcomes and was only partially accounted for by leave-one-out sensitivity analyses. All findings were rated as very low certainty according to the GRADE framework. **Conclusions**: Robotic-assisted splenectomy in pediatric patients has been reported as technically feasible and performed safely in selected cases. However, the small number of studies, their retrospective design, substantial methodological heterogeneity, and the resulting very low certainty of the evidence according to GRADE preclude any firm conclusions about its comparative safety or efficacy versus laparoscopy. Well-designed prospective studies are needed to clarify its clinical benefits.

## 1. Introduction

Laparoscopic splenectomy is considered the standard operative technique for malignant hematologic conditions and for selected non-malignant disorders, including immune thrombocytopenic purpura (ITP), sickle cell disease (SCD), and hereditary spherocytosis (HS) [1]. However, since it was first described in children, robotic-assisted splenectomy has emerged as a viable and increasingly attractive alternative for these indications [2]. In adult populations, multiple meta-analyses have reported that robotic splenectomy may lead to reduced intraoperative blood loss and fewer postoperative complications when compared to traditional laparoscopic methods [3]. Nevertheless, the use of robotic surgery in children has been somewhat limited, mainly due to challenges related to instrument dimensions, the smaller operative field, and a lack of widespread technical proficiency. Until recently, the literature on pediatric robotic splenectomy was sparse, mainly consisting of case reports and small patient series [4,5].

In this context, systematic reviews and meta-analyses evaluating robotic splenectomy outcomes in children are scarce. Previous studies have typically included small patient cohorts and presented inconsistent findings regarding clinical results and cost efficiency, partly due to the mixing of pediatric and adult populations and the considerable variability among cases [6,7]. However, over the past two to three years, there has been a notable increase in comparative studies focused exclusively on pediatric patients, reflecting growing expertise with this technique and highlighting the need for an updated and comprehensive review of the latest evidence [8,9,10]. This study sought to systematically review and synthesize the best comparative data currently available to evaluate and contrast the outcomes of robotic-assisted and laparoscopic splenectomy.

## 2. Methods

### 2.1. Search Strategy

This systematic review followed the guidelines outlined in the Preferred Reporting Items for Systematic Reviews and Meta-Analyses (PRISMA) [11,12]. The research question was formulated using the PICO (Population, Intervention, Comparator, Outcome) model: “What are the outcomes of robotic-assisted splenectomy in pediatric patients compared with conventional laparoscopic splenectomy?” A comprehensive literature search was conducted using four electronic databases: PubMed, CINAHL, Web of Science, and EMBASE. The initial search took place in July 2025, with an updated search performed on 1 August 2025, by two independent reviewers (CDM and JC). In PubMed, the search strategy included the following MeSH terms: [“robotic-assisted” OR “robotic”] AND [“children” OR “pediatric”] AND [“splenectomy” OR “spleen”]. The complete, database-specific search strategies are provided in Supplementary Material S1.

The primary aim was to determine the key outcomes of robotic-assisted splenectomy in the pediatric population, including surgical indications, patient demographics, operative duration, intraoperative complications, conversion to open surgery, long-term results, and the need for reintervention. As secondary objectives, the review sought to evaluate and compare these findings with those of laparoscopic splenectomy through a meta-analysis. The review protocol is registered with the PROSPERO database under the ID CRD420251104285.

### 2.2. Eligibility Criteria

Studies were deemed eligible for inclusion if they involved patients younger than 18 years and reported outcomes of robotic-assisted splenectomy (either partial or total) in this population. To be considered, articles had to be available in full text and contain original clinical data on robotic-assisted splenic procedures. Only studies involving human subjects and published in English were included. Exclusion criteria encompassed editorials, reviews without original data, commentaries, conference abstracts, animal studies, research limited to adult populations. Studies that included both pediatric and adult patients were only included if pediatric-specific data could be independently extracted. Duplicate records were eliminated, and titles and abstracts were initially screened to exclude those that did not clearly specify robotic-assisted splenectomy in pediatric patients. Full texts of potentially relevant articles were then thoroughly assessed to ensure alignment with the inclusion criteria. Review articles were temporarily retained to allow for manual reference list checks, applying a backward snowballing strategy to ensure comprehensive literature coverage. The screening process was conducted independently by two reviewers (CDM and JC), with discrepancies resolved through discussion until consensus was reached.

### 2.3. Data Collection

Relevant details from each selected study were extracted, including study design, number of participants, patient age at surgery, underlying diagnoses, type of surgical technique used, operation duration, conversion to open surgery, postoperative complications, and overall clinical outcomes. Two reviewers (CDM and JC) independently collected these data using a standardized spreadsheet in Microsoft Excel^TM^ (2007, Redmond, WA, USA). Data extraction was conducted separately by two reviewers (CDM and JC). Any discrepancies between reviewers were resolved through discussion until consensus was achieved. A descriptive synthesis was conducted to summarize the existing evidence on pediatric robotic-assisted splenectomy outcomes. As this study involved analysis of previously published data, ethical approval was not required.

### 2.4. Quality Appraisal and Risk of Bias

The methodological quality of the studies included was assessed using the Methodological Index for Non-Randomized Studies (MINORS) tool [13]. For the nonrandomized comparative studies included in the quantitative analysis, the Risk of Bias in Non-randomized Studies of Intervention (ROBINS-I) tool was used [14]. Risk of bias was assessed independently by two reviewers (CDM, JC). Any discrepancies between the reviewers were resolved by discussion with a third reviewer to reach an agreement.

### 2.5. Data Synthesis

Summary statistics reported as median and interquartile range (IQR) were converted to mean and standard deviation (SD) using the validated methods proposed by Wan et al. and Luo et al. [15,16]. The presence of skewness was assessed using the approach described by Shi et al. [17]. When significant skewness was identified, we applied the Quantile Estimation (QE) method developed by McGrath et al. [18].

### 2.6. Data Analysis

We conducted meta-analyses using random-effects models for both dichotomous and continuous outcomes. For continuous variables, pooled estimates were expressed as mean differences with 95% confidence intervals (CIs). For dichotomous outcomes, pooled effect measures were reported as risk ratios (RRs) or, when appropriate, as event rates per group according to the surgical technique. In cases where zero-event counts were present in one of the study arms (as occurred in the meta-analyses of conversion and complication rates), a modified Haldane–Anscombe continuity correction was applied. Heterogeneity across studies was assessed using the I^2^ statistic, the Cochran’s Q test, and the between-study variance (τ^2^). The observed heterogeneity, within-study variance, and the scale and distribution of the outcome informed model selection. Random-effects models were fitted by default using restricted maximum likelihood (REML) with Wald-type confidence intervals. To account for uncertainty in the estimation of between-study variance, particularly in analyses involving few studies, confidence intervals around the pooled estimates were adjusted using the Knapp–Hartung (HKSJ) or modified Knapp–Hartung (mKH) methods, following the Cochrane Handbook for Systematic Reviews of Interventions (version 6.5) [19]. When more than two studies were included and τ^2^ was non-zero, the HKSJ adjustment was generally preferred due to its superior statistical properties. In borderline scenarios, we conducted sensitivity analyses comparing HKSJ, mKH, and conventional Wald-type intervals, and reported the configuration offering the most robust and interpretable inference. In cases involving very few events, where HKSJ methods are known to perform poorly, REML estimation with conventional Wald-type intervals was used instead. When appropriate, we present results from multiple model configurations in parallel to ensure transparency and facilitate interpretability. Additionally, 95% prediction intervals (PIs) were calculated to provide an estimate of the expected range of effects in future settings. Given the small number of studies included in each analysis, neither visual (e.g., funnel plots) nor statistical assessments of small-study effects were performed, and meta-regression models were not developed due to limited power. An exploratory leave-one-out sensitivity analysis was conducted for each primary meta-analytical model to evaluate the influence of individual studies on the pooled estimates. All analyses were performed using Stata version 19.0 (StataCorp LLC, College Station, TX, USA) and R 4.5.1 as indicated.

### 2.7. GRADE Certainty of Evidence

The certainty of the evidence for each outcome was evaluated using the GRADE framework (Grading of Recommendations, Assessment, Development, and Evaluation) [20]. Each outcome was subsequently appraised across the standard GRADE domains—risk of bias, inconsistency, indirectness, imprecision, and publication bias—to determine whether downgrading or, where applicable, upgrading of the certainty rating was warranted.

## 3. Results

A total of 87 records were identified through database searches, from which 16 studies ultimately fulfilled the inclusion criteria and were incorporated into the qualitative synthesis of this systematic review. Of these, five studies were included in the quantitative analysis due to their comparative design between robotic and laparoscopic approaches. The analysis included 272 pediatric patients from studies published between 2003 and 2025. Among the included studies, 7 (43.7%) were retrospective descriptive analyses, 8 (31.3%) were retrospective comparative studies, 3 (18.8%) were case reports, and 1 (7.0%) was a prospective descriptive study, which are summarized in Table 1 and Table 2.

**Figure 1:**
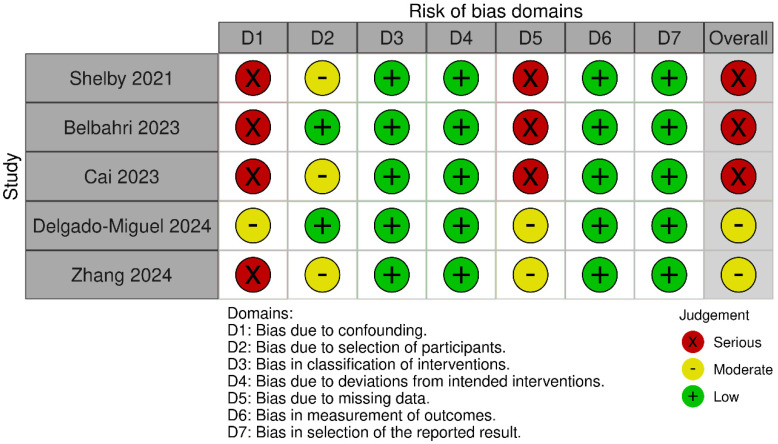
Risk of bias of the included studies using ROBINS-I tool.

**Figure 2:**
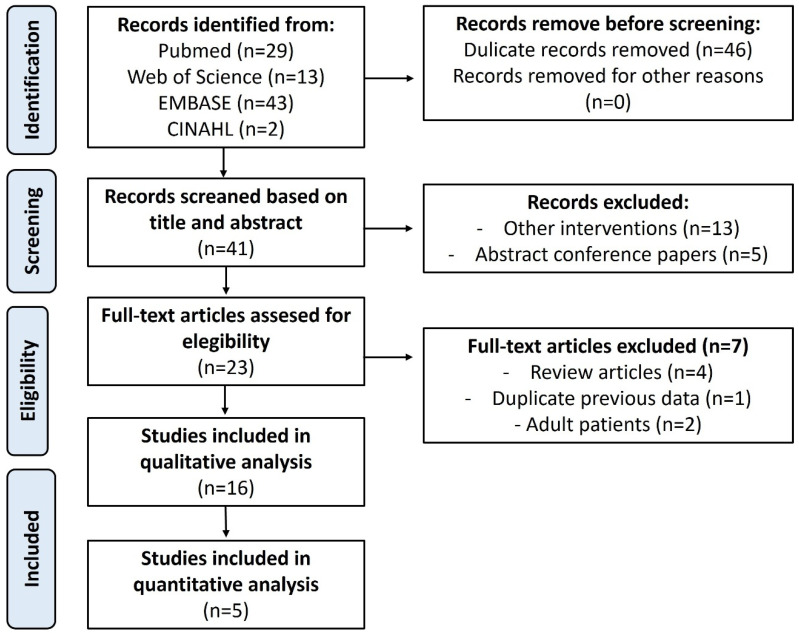
PRISMA flowchart.

Quality analysis according to the MINORS scale revealed the 5 comparative studies scoring 20 points (high quality), while the rest were between 14 and 16 points (low quality), corresponding to descriptive studies and clinical cases (Supplementary Material: Table S2). When analyzing the risk of bias among the comparative studies (Figure 1), three were at high risk of bias, and two studies were at moderate risk of bias, according to the ROBINS-I scale (Supplementary Material: Table S3). The process of article selection, along with the reasons for exclusion, is illustrated in the PRISMA flow diagram in Figure 2.

### 3.1. Qualitative Analysis

In 2003, Luebbe et al. reported the first two pediatric robot-assisted splenectomies in 5-year-old children with hypersplenism [2]. Both cases required conversion to open surgery due to bleeding at the splenic hilum, which impaired robotic visibility. The surgical cart was quickly undocked, and laparotomy performed without hemodynamic instability or need for transfusion. In 2007, both Najmaldin et al. and Klein et al. reported two successful robotic splenectomies each [4,21]. Later, Meehan et al. (2008) included eight robotic splenectomies among their first 100 robotic cases [22]. Four required conversion to open surgery due to bleeding from non-robotic issues such as a misfired stapler, trocar or grasper injury, and a misfired hemoclip. Alqahtani et al. reported 13 robotic cases within a series of 144 procedures [5]. Two required conversion to open surgery: one due to major bleeding needing urgent control, and another because the spleen’s large size prevented safe minimally invasive handling.

In 2020, Bisoffi et al. firstly used the Da Vinci Xi system to perform a splenectomy in a 15-year-old with chronic autoimmune thrombocytopenia [23]. Preoperative splenic artery embolization was done five days prior to surgery to raise platelet levels and reduce intraoperative bleeding risk, with no complications. Klazura et al. were the first to use the Da Vinci single-port robotic platform in 2021 for a 5-year-old girl with hemolytic anemia due to pyruvate kinase deficiency [24]. The single port was inserted under a cephalad skin flap midway to the umbilicus, through a Pfannenstiel incisión, that allowed successfully spleen removal intact (measuring 16.9 cm). Svetanoff et al. later used the same robotic system, placing the single-site port through a 2.5 cm vertical incision at the umbilicus in 11 patients [27]. For combined cholecystectomy and splenectomy, the gallbladder was removed first via the single port, and the robot was then undocked and repositioned, and two 8 mm additional ports were inserted. Del Conte et al. reported 12 splenectomies performed using the “Scarless Laparoscopic Incisions in Pfannenstiel” (SLIP) technique, in which trocars were placed along the Pfannenstiel line, that yielded favorable cosmetic outcomes with no reported complications [26].

In 2022, Khanna et al. used the Versius robotic system to perform a near-total cystectomy for a large splenic epidermoid cyst (15 × 12 cm), combined with a cholecystectomy in the same procedure, without intraoperative or postoperative complications [25]. Similarly, Mohady et al. reported the excision of a splenic dermoid cyst as part of their series of nine abdominal tumors treated robotically [28]. Vasilescu et al. compared 10 robotic and 22 laparoscopic subtotal splenectomies, including 26 pediatric and six adult patients, with a mean age of 13.15 years (range 5–35 years) [29]. Due to the lack of disaggregated pediatric data, this study could not be included in the present analysis. Similarly, Manci et al. compared outcomes of subtotal laparoscopic and robotic splenectomy versus total splenectomy in 63 patients with hereditary spherocytosis. However, since the cohort included both adults and children without age-specific stratification, the study was not eligible for inclusion in the quantitative analysis of the present review [30]. Notably, the heterogeneity in patient characteristics across studies—particularly in age, weight, and spleen pathology—suggests that surgical outcomes may be closely linked to individual anatomical and physiological factors. This finding reinforces the need for a personalized surgical approach when selecting candidates for robotic splenectomy.

### 3.2. Quantitative Analysis

#### 3.2.1. Operative Time

**Figure 3:**
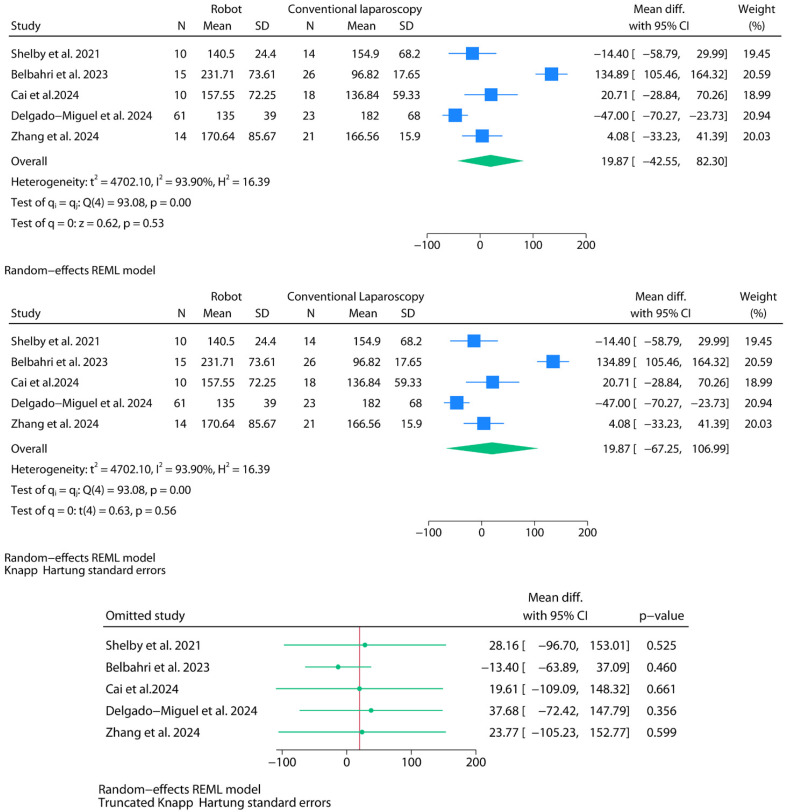
Meta-analysis of operative time (in minutes).

Operative time (in minutes) was meta-analyzed using data from five studies (Figure 3). A random-effects model was applied using REML estimation and Wald-type confidence intervals. The pooled mean difference between robotic and laparoscopic approaches was 19.87 min (95% CI: −42.55 to 82.30; *p* = 0.53). Between-study heterogeneity was extreme, with I^2^ = 93.9% and Cochran’s Q = 93.08 (*p* < 0.01). The estimated between-study variance was substantial (τ^2^ = 4702.10). A subsequent adjustment using the HKSJ method widened the confidence interval (95% CI: −67.25 to 106.99). It slightly increased the model *p*-value (*p* = 0.56) but did not materially alter the inference. The 95% prediction interval ranged from −220.74 to 260.49 min. Leave-one-out sensitivity analysis using truncated HKSJ-adjusted intervals showed no exclusion that shifted the result toward statistical significance. However, the removal of Belbahri et al.—a study reporting unusually long operative times for robotic surgery and exceptionally short times for laparoscopy—had the most significant numerical impact, shifting the mean difference modestly in favor of robotic surgery (−13.40 min; 95% CI: −63.89 to 37.09), although still not reaching statistical significance [8]. Notably, this exclusion was also associated with a marked reduction in heterogeneity (I^2^ = 64.2%) and a corresponding drop in Cochran’s Q (*p* = 0.03), revealing that this study was a significant source of between-study variability.

**Table 2:** Studies included in quantitative synthesis (meta-analysis).

References (Country, Year)	Study Design	No. Cases	Age (Years)	Robot Model	Operating Time (min) *	Postoperative Complications	Conversion to Open	Hospital Stay (Days) *
**Shelby et al. (USA, 2021)** [31]	Retrospective comparative	24 Rob: 10 Lap: 14	Rob: 9.9 (SD: 4.5) Lap: 11.3 (SD: 6.3)	Si	Rob: 140.5 (SD: 24.4) Lap: 154.9 (SD: 68.2)	Rob: 2 Lap: 1	Rob: 1 Lap: 0	Rob: 2.1 (1–2.4) Lap: 3.2 (2.5–4.2)
**Belbahri et al. (France, 2023)** [8]	Retrospective comparative	41 Rob: 15 Lap: 26	Non-specified	Non-specified	Rob: 223 (190–280) Lap: 97 (85.5–108)	Non-specified	Rob: 0 Lap: 0	Rob: 5 (5–5.5) Lap: 6.5 (5–8)
**Cai et al. (China, 2023)** [10]	Retrospective comparative	32 Rob: 13 Lap: 19	Rob: 12 (9.6–15.5) Lap: 10.8 (9–13.7)	Xi	Rob: 140 (120–207.5) Lap: 140 (98.75–172.5)	Rob: 0 Lap: 1	Rob: 0 Lap: 4	Rob: 11 (7.75–13.5) Lap: 9.5 (7.75–13)
**Delgado-Miguel et al. (USA, 2024)** [9]	Retrospective comparative	84 Rob: 61 Lap: 23	Rob: 8.2 (5.7–12.2) Lap: 10 (3.8–15.3)	Si	Rob: 135 (SD: 39) Lap: 182 (SD: 68)	Rob: 1 Lap: 4	Rob: 0 Lap: 0	Rob: 3 (2–3) Lap: 3 (2–4)
**Zhang et al. (China, 2024)** [32]	Retrospective comparative	35 Rob: 14 Lap: 21	Rob: 10.1 (9.1–11.1) Lap: 8.6	Xi	Rob: 167 (120–224) Lap: 168 (156–176)	Rob: 1 Lap: 3	Rob: 0 Lap: 3	Rob: 8 (7–9.3) Lap: 10 (9–12)

* Intervals refer to the interquartile range (IQR); SD, standard deviation; USA, United States of America.

#### 3.2.2. Length of Hospital Stay

In the study by Belbahri et al., hospital stay in the robotic group was reported as a median of 5 days (IQR: 5–5.5) [8]. Given the minimal spread of the interquartile range (with Q1 equal to the median), applying a transformation method to estimate the mean and standard deviation was technically challenging. In this particular case, the use of the McGrath method likely yields a slightly conservative estimate of the standard deviation; however, we consider this preferable to ensure a uniform and transparent handling of data across studies. A similar situation occurred in the study by Delgado-Miguel et al., in which the hospital stay was reported as a median of 3 days (IQR: 2–3; n = 61) for the robotic splenectomy group [9]. Again, due to the compressed interquartile range, the estimated standard deviation may slightly overstate the true variability; however, a consistent conversion method was used across all studies.

**Figure 4:**
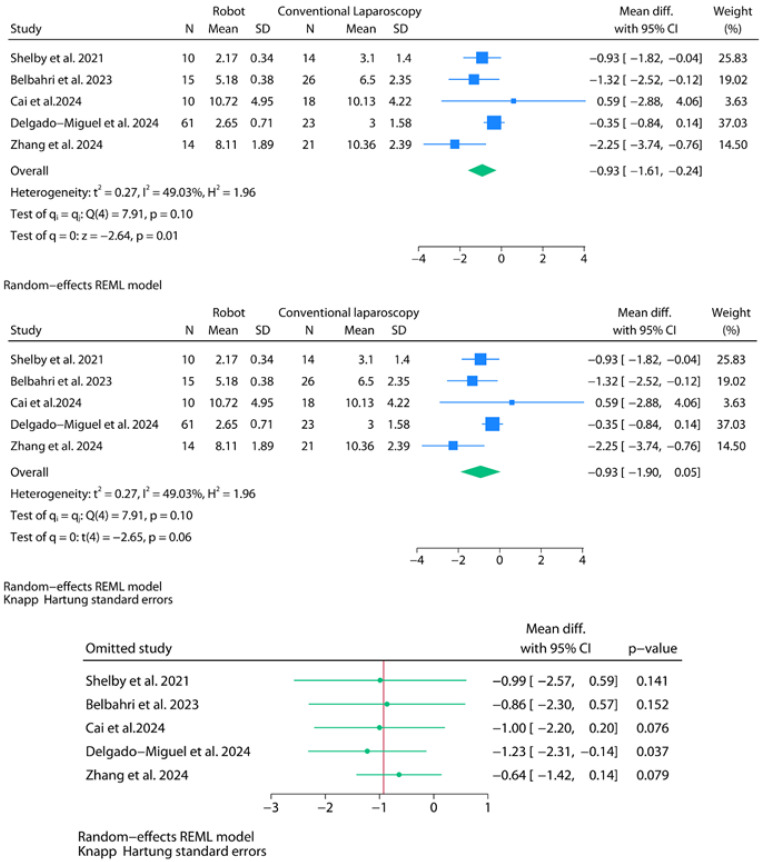
Meta-analysis of hospital stay (in days).

Five studies were included in the meta-analysis of hospitalization time (in days). A random-effects model was fitted using the REML estimator with default Wald-type confidence intervals (Figure 4). The pooled mean difference between robotic and laparoscopic approaches was −0.93 days (95% CI: −1.61 to −0.24; *p* = 0.01), indicating significantly shorter stays with robotic surgery. Between-study heterogeneity was moderate (I^2^ = 49.0%), with Cochran’s Q = 7.91 (*p* = 0.10), and the estimated between-study variance was τ^2^ = 0.27. A subsequent adjustment using the HKSJ method widened the confidence interval (95% CI: −1.90 to 0.05) and attenuated the statistical significance of the model to marginal levels (*p* = 0.06). The 95% prediction interval ranged from −3.4 to 2.0 days. Leave-one-out sensitivity analysis using HKSJ-adjusted intervals revealed that excluding Delgado-Miguel et al. had the greatest impact, improving the model in favor of significantly shorter stays with robotic surgery (−1.23 days; 95% CI: −2.31 to −0.14; *p* = 0.037). This influence is explained by the relatively large weight of this study in the REML model (37.0%), derived from its high sample size, and by the small inter-group differences combined with a higher standard deviation in the laparoscopic group. In contrast, the exclusion of Zhang et al.—a study characterized by prolonged hospital stays and wide confidence intervals due to an imputed mean—was the one that showed the least favorable outcomes for the robotic group [32].

#### 3.2.3. Intraoperative Blood Loss

**Figure 5:**
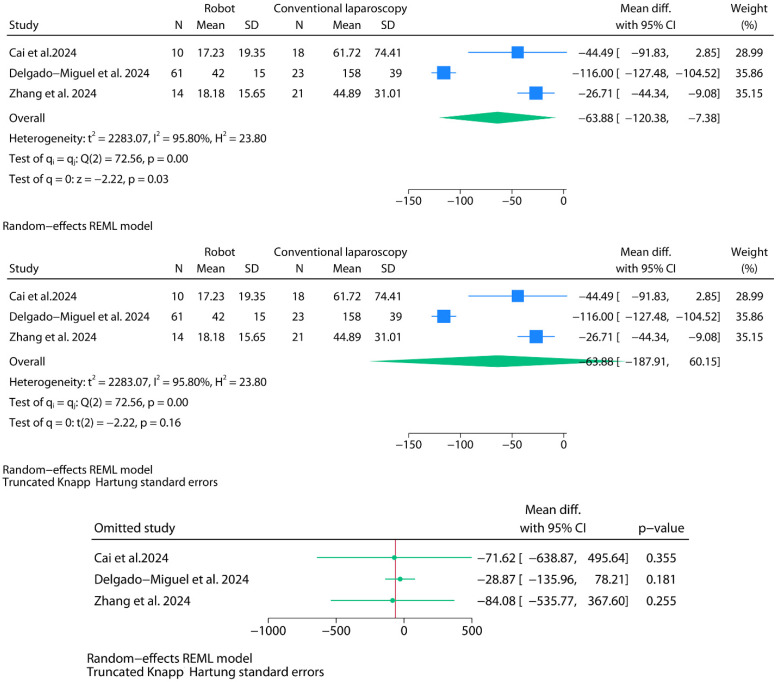
Meta-analysis of blood loss (in milliliters).

For the meta-analysis of intraoperative blood loss (in milliliters), only three studies contributed usable data (Figure 5). A random-effects model was fitted using REML with default Wald-type confidence intervals. The pooled mean difference between robotic and laparoscopic approaches was −63.88 mL (95% CI: −120.38 to −7.38; *p* = 0.03), favoring reduced blood loss in the robotic group. Between-study heterogeneity was extreme (I^2^ = 95.8%), with Cochran’s Q = 72.56 (*p* < 0.001), and the between-study variance was substantial (τ^2^ = 2283.07). A subsequent adjustment using the truncated Knapp–Hartung (mKH) method widened the confidence interval (95% CI: −187.91 to 60.15) and rendered the statistical significance null (*p* = 0.16). What stands out most in this analysis is the particularly high blood loss reported in the laparoscopic group by Delgado-Miguel et al. [9], and the wide dispersion observed in the study by Cai et al. [10], which is likely attributable to the significant standard deviation resulting from the conversion of summary statistics. These factors—together with the small number of studies, extreme heterogeneity, and the intrinsic difficulty of accurately measuring intraoperative blood loss in milliliters—warrant considerable caution in interpreting this result. The 95% prediction interval ranged from −772.93 to 645.17 mL. Leave-one-out sensitivity analysis using mKH-adjusted intervals did not identify any exclusion that materially altered the direction or significance of the pooled estimate.

#### 3.2.4. Conversion Rate

**Figure 6:**
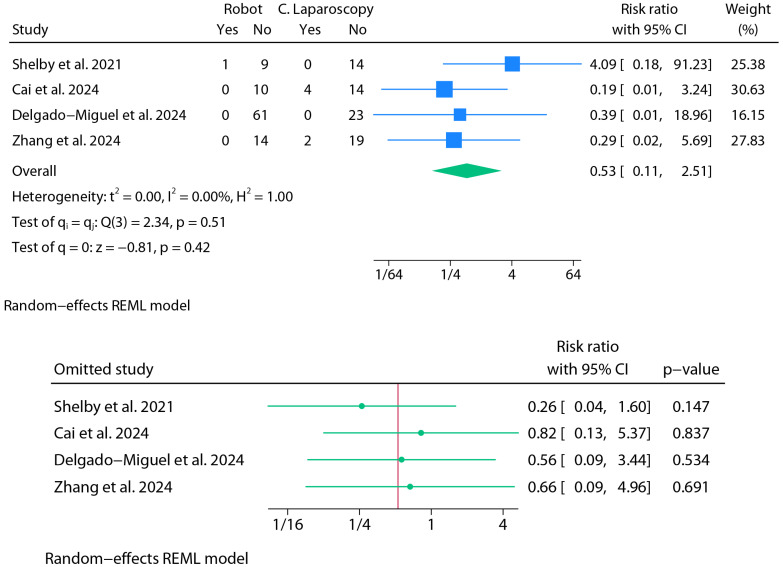
Meta-analysis of conversions to open surgery.

For the meta-analysis of conversions to open surgery, four studies contributed usable binary data (Figure 6). A random-effects model was fitted using REML without Knapp–Hartung adjustments. The pooled RR comparing robotic versus laparoscopic approaches was 0.53 (95% CI: 0.11 to 2.51; *p* = 0.42). Statistical heterogeneity was null (I^2^ = 0.0%), with Cochran’s Q = 2.34 (*p* = 0.51), and the estimated between-study variance was negligible (τ^2^ = 0.00). The 95% prediction interval (RR) ranged from 0.017 to 16.292. Leave-one-out sensitivity analysis under the REML model showed that excluding Shelby et al. [31] yielded the largest numerical shift, further favoring the robotic group (RR = 0.26; 95% CI: 0.04 to 1.60; *p* = 0.147), though the result still did not reach statistical significance. This illustrates how a single event—such as the conversion reported in Shelby et al.—can markedly affect the pooled estimate in studies with very small sample sizes. Accordingly, these results must be interpreted with caution. Belbahri et al. reported a single case of conversion to open surgery [8]; however, the report did not specify whether this event occurred in the robotic or laparoscopic group. As a result, the data could not be incorporated into the comparative analysis of conversion rates.

#### 3.2.5. Reinterventions

For the outcome of reintervention, only three studies reported relevant data. Across these, all treatment arms had zero events, except for a single event occurring in the laparoscopic group in one study. Given the extremely low event rate and the presence of zero-cell counts in nearly all comparisons, conducting a meta-analytic synthesis was deemed statistically uninformative. Under such sparse conditions, pooled effect estimates would be unstable and potentially misleading, offering little added value over a descriptive summary of the raw data.

#### 3.2.6. Postoperative Blood Transfusions

**Figure 7:**
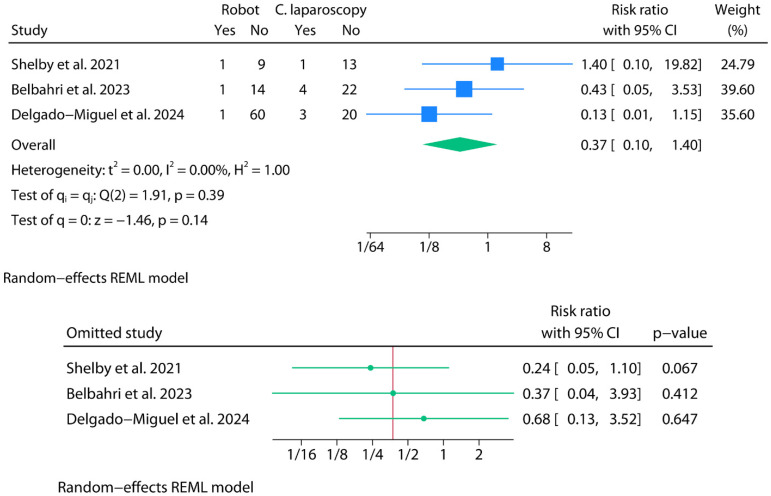
Meta-analysis of blood transfusions.

For the meta-analysis of postoperative blood transfusions, only three studies contributed usable binary data (Figure 7). A random-effects model was fitted using REML without Knapp–Hartung adjustments, given the poor performance of HKSJ-type corrections in meta-analyses with very few events and null τ^2^. The pooled RR comparing robotic versus laparoscopic approaches was 0.37 (95% CI: 0.10 to 1.40; *p* = 0.14). Statistical heterogeneity was null (I^2^ = 0.0%), with Cochran’s Q = 1.91 (*p* = 0.39), and the estimated between-study variance was also negligible (τ^2^ = 0.00). The 95% prediction interval (RR) ranged from 0.00 to 1938.17. Leave-one-out sensitivity analysis under the REML model revealed that excluding Shelby et al. [30] yielded the greatest shift, rendering the pooled effect marginally significant (RR = 0.29; 95% CI: 0.09 to 0.94; *p* = 0.07). However, given the limited number of studies, the very low event rates, and the small total sample size, this result should be interpreted with extreme caution. Belbahri et al. reported postoperative blood transfusion rates of 7.1% in the robotic group and 15.1% in the laparoscopic group [8]. Although exact denominators were not provided, the study indicated that approximately 15 patients underwent robotic surgery and 26 underwent laparoscopic surgery. Based on these estimates, the rates likely correspond to approximately one event in the robotic group and four in the laparoscopic group. Due to the absence of precise sample sizes, these counts should be interpreted with caution. It should be noted that a substantial proportion of patients undergoing splenectomy, particularly in these cohorts, present with underlying hematological conditions. In this context, the need for blood transfusion should not be automatically equated with a perioperative complication, as it may reflect baseline disease severity rather than surgical adverse events. Accordingly, transfusion data must be interpreted with caution and in light of the patients’ underlying clinical profiles.

#### 3.2.7. Postoperative Complications

For this review, complications were classified as intraoperative (adverse events occurring during the surgical procedure, such as bleeding requiring conversion or transfusion), postoperative (events arising after completion of the procedure, such as infections or hydrops), and perioperative (aggregate categories reported in some studies that included both intra- and postoperative events, as well as non-surgical occurrences such as thromboembolic or respiratory complications). This classification was adopted to harmonize the heterogeneous reporting across studies. Inevitably, some overlap remained, particularly for conversions due to intraoperative bleeding, which several primary reports described simultaneously as “conversions” and as “complications”. In line with the original data presentation, these events were included under both outcomes to ensure comparability. Still, this approach should be interpreted with caution given the potential for double counting.

Belbahri et al. [8] reported operative and postoperative complications in aggregate form, without providing a clear breakdown of event counts per surgical group. For example, while they state an overall complication rate of 4.9% and describe several specific events—such as bleeding leading to conversion, use of hemostatic agents, transfusions, and Clavien–Dindo < 2 complications—these are not consistently attributed to either the robotic or laparoscopic group. As a result, it was not possible to extract group-specific event data suitable for inclusion in the meta-analysis.

In the study by Shelby et al. [31], complications were reported as part of a broader category of “perioperative occurrences”, which included not only surgical events but also non-surgical conditions such as deep venous thrombosis and respiratory complications. Notably, surgical conversions were also grouped within this category. As a result, the conversion event, already accounted for in the separate meta-analysis of conversion rates, is effectively counted twice in this outcome. Since this was the only format in which perioperative data were reported, it was the only configuration available for meta-analysis. However, due to the heterogeneous nature of the events included and the overlap with other outcomes, these results should be interpreted with caution.

In the study by Cai et al. [10], four conversions to open surgery were reported in the laparoscopic group, all attributed to intraoperative bleeding. While these events were already included in the meta-analysis of conversion rates, they were also counted as surgical complications in the present analysis, given their direct link to hemorrhagic events. No complications were clearly reported for the robotic group, and the single case of postoperative encapsulated hydrops was not assigned to either group and was therefore not considered. As complications were not reported in a standardized or disaggregated format, and given the overlap with other outcomes, the data from this study were incorporated cautiously and should be interpreted with appropriate methodological reservations.

Zhang et al. stratified their reported complications into preoperative and postoperative events [32]. To maintain internal consistency across studies, and following the same criterion applied to Shelby et al. [31]—where perioperative occurrences encompassed both intra- and postoperative events—we included both categories in the meta-analysis as a single aggregated complication outcome. Notably, the three intraoperative complications reported in the laparoscopic group corresponded to bleeding events that led to conversion to open surgery, mirroring the scenario described in Cai et al. [10]. This justified counting them both as complications and as conversions. In contrast, the robotic group in Zhang et al. [32] reported no intraoperative complications; the only complication occurred in the postoperative period. This approach ensures uniformity in the definition of complications across studies, although it may slightly broaden the scope of what is considered a surgical complication.

**Figure 8:**
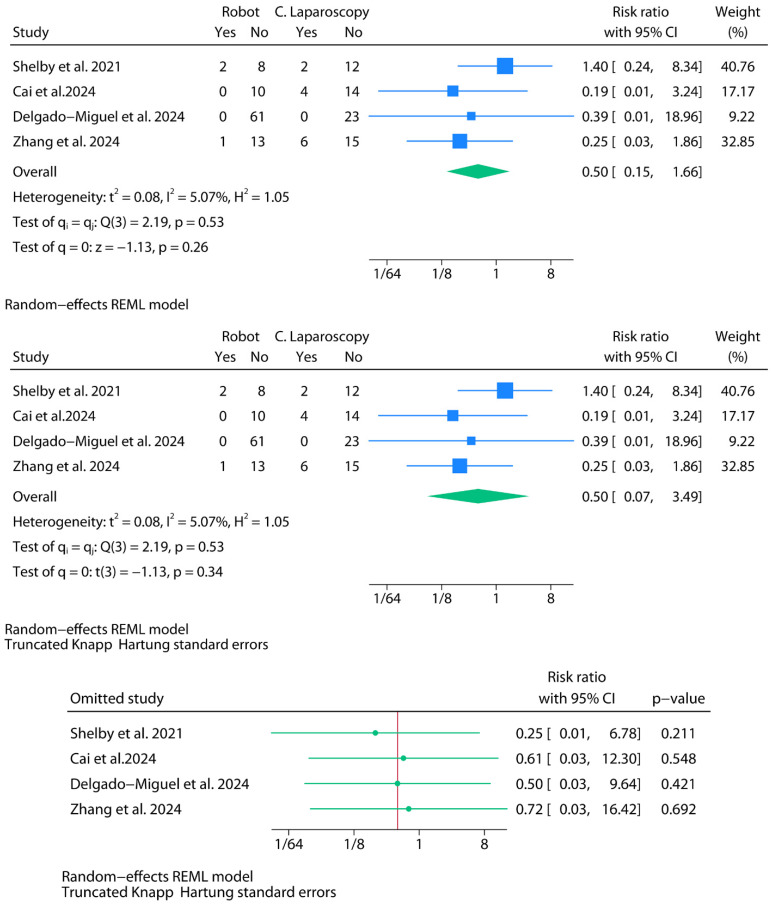
Meta-analysis of postoperative complications.

For the meta-analysis of perioperative complications, four studies contributed usable binary data (Figure 8). A random-effects model was fitted using REML. The pooled risk ratio comparing robotic versus laparoscopic approaches was 0.50 (95% CI: 0.15 to 1.66; *p* = 0.26). Statistical heterogeneity was very low (I^2^ = 5.1%), with Cochran’s Q = 2.19 (*p* = 0.53), although the estimated between-study variance was not null (τ^2^ = 0.08). Under mKH-adjusted intervals, the confidence interval widened to 0.07 to 3.49, and the model *p*-value increased to 0.34. The 95% prediction interval (RR) ranged from 0.03 to 9.05. As a sensitivity analysis, we replicated the meta-analysis after removing conversions from the complication outcome. This adjustment yielded a pooled RR of 0.83 (95% CI: 0.21–3.23; *p* = 0.79), shifting the estimate towards the null and substantially attenuating the apparent advantage of the robotic approach.

Leave-one-out sensitivity analysis using mKH-adjusted intervals did not reveal any exclusion that materially changed the pooled result. The exclusion of Shelby et al.—a study that proportionally reported more complications in the robotic group—produced a modest improvement in the overall effect estimate favoring the robotic approach, though the result remained statistically non-significant. This pattern could be interpreted or justified by the fact that Shelby et al. represent one of the earliest published experiences in this setting, potentially reflecting limited collective experience with the robotic approach at the time, including refinement of surgical techniques, as well as the use of earlier-generation robotic platforms with more restricted technical capabilities. Nevertheless, this interpretation remains speculative and is not directly supported by the primary data.

### 3.3. GRADE Assessment and Certainty of Evidence

Across all outcomes, the certainty of evidence was rated as very low. Because all included studies were retrospective cohorts rather than randomized controlled trials, the certainty of evidence started at a low level under the GRADE framework and was further downgraded for multiple reasons. Treatment allocation was uncontrolled, and important prognostic factors (e.g., spleen size, underlying pathology, or surgeon experience) may have influenced both the surgical approach and the observed outcomes. Imprecision was a major concern for several endpoints, as extremely wide confidence intervals frequently spanned potential benefit and harm, reflecting small sample sizes and sparse event counts. For outcomes such as operative time and blood loss, heterogeneity was extreme and remained largely unexplained despite sensitivity analyses. In the case of perioperative complications, additional concerns arose from inconsistent definitions across studies and a unit-of-analysis error that artificially inflated events in one arm. Taken together, these limitations markedly constrain the interpretability of the pooled estimates and preclude any firm conclusions regarding the comparative performance of the two techniques.

## 4. Discussion

This systematic review and meta-analysis provided a comprehensive assessment of the existing evidence on robotic splenectomy techniques in the pediatric population in the last 20 years. This study focused on qualitatively and quantitatively assessing the outcomes of robotic splenectomy in children, a group in which this procedure is frequently indicated for several non-traumatic conditions, particularly hematologic disorders. Early reports, such as those by Luebbe et al. [2], highlighted challenges related to bleeding control, often leading to conversion to open surgery. However, subsequent series demonstrated increased procedural safety and feasibility as experience and robotic technology improved [4,21,22]. Several authors have emphasized the advantages of enhanced visualization and precision in dissection, particularly around the splenic hilum, as benefits of the robotic approach [8,9]. The use of adjunct techniques—such as preoperative splenic artery embolization or transumbilical single-port platforms—was associated with improved cosmetic outcomes and potentially reduced intraoperative risks [24,26]. While most procedures were completed without major complications, a few required conversion due to technical difficulties or uncontrolled bleeding, although these events decreased over time. Overall, the qualitative findings suggest that robotic splenectomy in pediatric patients is safe and feasible in selected cases, with potential advantages in complex dissections and cosmetic outcomes, and could contribute to the broader framework of personalized pediatric surgery by allowing surgeons to adapt the technique to the individual patient’s anatomy, pathology, and perioperative needs. However, more standardized, prospective studies are needed to draw definitive conclusions.

In recent years, robotic splenectomy has been increasingly compared to the laparoscopic approach in terms of intraoperative variables (operative time, intraoperative bleeding, need for conversion to open surgery) and postoperative outcomes (requirement for transfusion, surgical reinterventions, postoperative complications). However, the available literature to date is methodologically limited and difficult to extrapolate to the pediatric population. The findings of a published meta-analysis in adult patients [3] suggested that the robotic splenectomy is associated with fewer post-operative complications and conversion to an open procedure than the laparoscopic approach. However, the findings of the meta-analysis mentioned above are of doubtful merit due to selective reporting, suboptimal analyses, and erroneous data extraction. In 2022, Bhattacharya et al. performed another meta-analysis in adult patients, which included a subgroup of pediatric patients with only two studies, with inconclusive results [6]. Shortly thereafter, in the same year, Ghidini et al. published the first systematic review on the outcomes of robotic-assisted splenectomy exclusively in pediatric patients [7]. They performed a qualitative analysis that included five studies with a total of nine patients, while their quantitative analysis compared outcomes between laparoscopic and robotic-assisted splenectomy in children. However, among the five studies included in the meta-analysis, only three directly compared the laparoscopic and robotic approaches, and one of them combined both pediatric and adult patients in the analysis [29]. During the last few years, the increased availability of robotic surgical systems and broader institutional access to robotic-assisted surgery have contributed to a substantial rise in the number of pediatric cases treated with robotic-assisted surgery. This trend is reflected in the growing number of scientific publications over the past three years comparing laparoscopic and robotic splenectomy techniques in children, which has significantly expanded the existing body of evidence on the topic [9,10,32]. Collectively, these factors support the rationale for conducting an updated review of the current evidence regarding this subject in pediatric patients.

In this context, the present study provides an updated overview of the accumulated experience with robotic splenectomy in pediatric patients, including a total of 272 cases, of which 169 were performed using a robotic approach. While the number of cases remains limited compared to adult series, it still permits a reasonably precise qualitative and quantitative evaluation. Overall, robot-assisted splenectomy can be considered a safe and effective surgical technique. A total of nine conversions to open surgery were reported (5.3%); four were unrelated to robotic system issues, while the remaining five were due to intraoperative bleeding or excessive spleen size, which hindered minimally invasive management. Our review employed robust statistical methodology, including the use of random-effects models fitted via REML, with confidence intervals adjusted using either the mKH or HKSJ approaches—both recommended in contemporary methodological guidance, particularly when dealing with meta-analyses based on a limited number of studies and high heterogeneity. Sensitivity analyses were systematically conducted through exploratory leave-one-out procedures, allowing for a structured exploration of study-level influence and potential sources of heterogeneity. However, this review is constrained by several key limitations. First, the number of included studies per outcome was frequently low, and individual sample sizes were often modest, thereby limiting statistical power and interpretability. In some outcomes—such as hospital readmissions—meta-analytic pooling was not feasible due to insufficient or inconsistently reported data. Moreover, in several models, the heterogeneity observed was substantial to extreme, undermining the certainty of pooled estimates and complicating the extraction of clinically meaningful conclusions. The use of continuity corrections, although widely implemented in practice and rarely declared explicitly, represents an inferential limitation that partially alters the original data and necessitates cautious interpretation of the results [33]. In addition, handling zero-event counts, few-event studies, and skewed interquartile range conversions may have introduced potential biases despite the use of rigorous, evidence-based, and transparently reported methods. It should also be noted that in meta-analyses with very few studies (*k* = 3), as in this case, the estimation of between-study heterogeneity is particularly challenging. While REML generally performs better than traditional approaches such as the DerSimonian–Laird method, it is still not flawless and may underestimate the true variability. In this context, Wald-type confidence intervals tend to be overly optimistic, whereas corrections such as HKSJ or its modified version (mHK) can become overly conservative. When the number of studies is very limited, especially with sparse events, these methods face inherent limitations, and all results should therefore be interpreted with great caution. In the present review, the included meta-analyses also displayed substantial heterogeneity, resulting in very wide 95% prediction intervals. This, combined with the retrospective nature of the primary studies, means that, according to the GRADE framework, all pooled findings must be rated as very low certainty.

Although several potentially relevant variables were extracted to perform meta-regression analyses—such as whether the same surgeon performed both laparoscopic and robotic procedures, whether the splenectomy was total or subtotal, the presence of significant differences in spleen size between groups, and the distribution of underlying pathologies across surgical arms—the limited number of included studies, together with the presence of missing data and the predominantly polychotomous nature of these covariates, rendered formal meta-regression statistically unjustifiable due to low power and high risk of spurious findings. Nonetheless, these variables have been retained in the underlying dataset to facilitate future meta-analytical modeling as the number of eligible studies increases. An additional consideration is the heterogeneity of robotic platforms used across the included studies, ranging from early systems such as ZEUS to more recent generations, including Da Vinci Xi, Da Vinci SP, and Versius. Earlier systems offered more limited dexterity, imaging, and instrumentation, which may have contributed to longer operative times and higher conversion rates reported in older studies. In contrast, newer platforms incorporate enhanced visualization, advanced energy devices, and improved instrument versatility, potentially translating into greater efficiency and safety in pediatric procedures. However, due to the small sample sizes and heterogeneous reporting of outcomes, no definitive conclusions can be drawn regarding the impact of specific robotic platforms at present.

Beyond platform heterogeneity, it is also important to acknowledge that the overall null findings of this review may partly reflect the averaging of studies conducted across different technological eras and stages of institutional learning. Pooling early experiences with more contemporary series introduces an important, yet essentially unquantifiable, source of clinical heterogeneity. Acknowledging this narrative–data disconnect adds a critical interpretative layer and highlights that future research should specifically evaluate current-generation robotic systems, ideally once institutional learning curves have been completed under standardized reporting conditions.

## 5. Conclusions

In summary, the current evidence on robotic-assisted splenectomy in pediatric patients remains extremely limited, heterogeneous, and of very low certainty. No statistically significant differences were demonstrated across most outcomes; however, the consistent direction of effect estimates favoring the robotic approach—particularly for blood loss, hospital stay, and conversion or complication rates—may indicate a potential clinical signal. These trends, nevertheless, must be interpreted with considerable caution due to the small number of studies, methodological variability, and overall low-quality evidence. The methodological rigor applied, including validated data transformations and conservative statistical adjustments, enhances the internal validity of the analysis but cannot compensate for the fundamental limitations of the evidence base. While the procedure appears technically feasible and has not raised unexpected safety concerns in published series, the available data remain insufficient to draw reliable conclusions regarding its comparative effectiveness or safety relative to laparoscopy. Well-designed, prospective comparative studies with standardized outcome definitions and adequate sample sizes are urgently needed before the clinical value of the robotic approach can be meaningfully established. Until such evidence becomes available, robotic splenectomy should be considered a promising yet still investigational alternative in this setting.

## Figures and Tables

**Table 1:** Studies included in qualitative analysis.

References (Country, Year)	Study Design	No. Cases	Age (Years)	Robot Model	Operating Time (min)	Postoperative Complications	Conversion to Open	Hospital Stay (Days)
**Luebbe et al. (USA, 2003) [2]**	Retrospective descriptive	2	5 (both)	S	128 (19–394) *	None	2	2.7 (range: 1–16) *
**Najmaldin et al. (UK, 2007) [4]**	Retrospective descriptive	2	10.2 ± 4.1 *	Non-specified	233 and 287	1 (wound infection)	None	3
**Klein et al. (USA, 2007) [21]**	Retrospective descriptive	2	Non-specified	Zeus	Non-specified	None	None	Non-specified
**Meehan et al. (USA, 2008) [22]**	Retrospective descriptive	8	8.4 *	S	105 (range: 53–730) *	None	4	Non-specified
**Alqahtani et al. (Saudi Arabia, 2010) [5]**	Retrospective descriptive	13	8.9 *	S	165 (range: 50–300) *	None	2	Non-specified
**Bisoffi et al. (Italy, 2020) [23]**	Case report	1	15	Xi	280	None	None	Non-specified
**Klazura et al. (USA, 2021) [24]**	Case report	1	5	SP	164	None	None	4
**Khanna et al. (India, 2023) [25]**	Case report	1	13	Versius	100	None	None	2
**Del Conte et al. (France, 2023) [26]**	Prospective descriptive	14	6 (range: 2.5–15)	Xi	134.5 (range: 77–165)	None	None	2 (range: 2–4)
**Svetanoff et al. (USA, 2024) [27]**	Retrospective descriptive	11	14.9 (range: 7.2–17.2)	SP	227 min (IQR 115, 318)	1 (hematoma in splenic bed)	None	2.1 (IQR 2.0–3.1)
**Mohady et al. (2025) [28]**	Retrospective descriptive	1	14 (range: 8–17) *	Si	Non-specified	None	None	Non-specified

* Data refer to the total series of patients (not exclusively splenectomies); UK, United Kingdom; USA, United States of America; IQR, interquartile range; SP, Single-Port.

## Data Availability

Data collection templates, extracted datasets, and other study materials are available upon reasonable request from the corresponding author. This systematic review is registered in the PROSPERO database (https://www.crd.york.ac.uk/PROSPERO/view/CRD420251104285 accessed 11 August 2025).

## Supplementary Materials

The following supporting information can be downloaded at: https://www.mdpi.com/article/10.3390/jpm15110522/s1, Supplementary Material S1: search strategies for each data-base; Supplementary Material S2: Dataset MA splenectomy; Supplementary Tables S1: MI-NORS_scores Robotic Splenectomy; Supplementary Tables S2: ROBINS_I_splenectomy.
