# Supplementary material for: Robotic-Assisted vs. Laparoscopic Splenectomy in Children: A Systematic Review and Up-to-Date Meta-Analysis
Source: J Pers Med. 2025 Nov 1;15(11):522. doi: 10.3390/jpm15110522 (PMC12653831; doi:10.3390/jpm15110522)
Supplement: Supplementary file 1 [file jpm-15-00522-s001.zip › Supplementary Material S1. Search strategy.pdf]

1. PubMed/MEDLINE

- **Search date:** August 1, 2025
- **Search equation:** (("robotic-assisted"[All Fields] OR "robotic"[All Fields]) AND ("children"[MeSH Terms] OR "pediatric"[All Fields]) AND ("splenectomy"[MeSH Terms] OR "spleen"[All Fields]))
- **Filters applied:** Language: English; Species: Humans
- **Results:** 29

2. Web of Science (Core Collection)

- **Search date:** August 1, 2025
- **Search equation:** TS=("robotic-assisted" OR "robotic") AND TS=("children" OR "pediatric") AND TS=("splenectomy" OR "spleen")
- **Filters applied:** Language: English; Document type: Articles
- **Results:** 13

3. Embase

- **Search date:** August 1, 2025
- **Search equation:** ('robot assisted':ab,ti OR robotic:ab,ti) AND ('child'/exp OR pediatric:ab,ti) AND ('splenectomy'/exp OR spleen:ab,ti)
- **Filters applied:** Language: English; Humans
- **Results:** 43

4. CINAHL (EBSCOhost)

- **Search date:** August 1, 2025
- **Search equation:** ("robotic-assisted" OR "robotic") AND ("children" OR "pediatric") AND ("splenectomy" OR "spleen")
- **Filters applied:** Language: English; Humans; Document type: Academic articles
- **Results:** 2
